# Evaluation of Natural Organic Additives as Eco-friendly Inhibitors for Calcium and Magnesium Scale Formation in Water Systems

**DOI:** 10.1021/acsenvironau.4c00076

**Published:** 2024-10-14

**Authors:** Amthal Al-Gailani, Martin J. Taylor, Muhammad Hashir Zaheer, Richard Barker

**Affiliations:** † School of Engineering, Chemical Engineering, University of Hull, Hull HU6 7RX, United Kingdom; ‡ School of Mechanical Engineering, University of Leeds, Leeds LS2 9JT, United Kingdom

**Keywords:** mineral precipitation, calcium carbonate, magnesium deposits, organic additives, inhibitor, adsorption

## Abstract

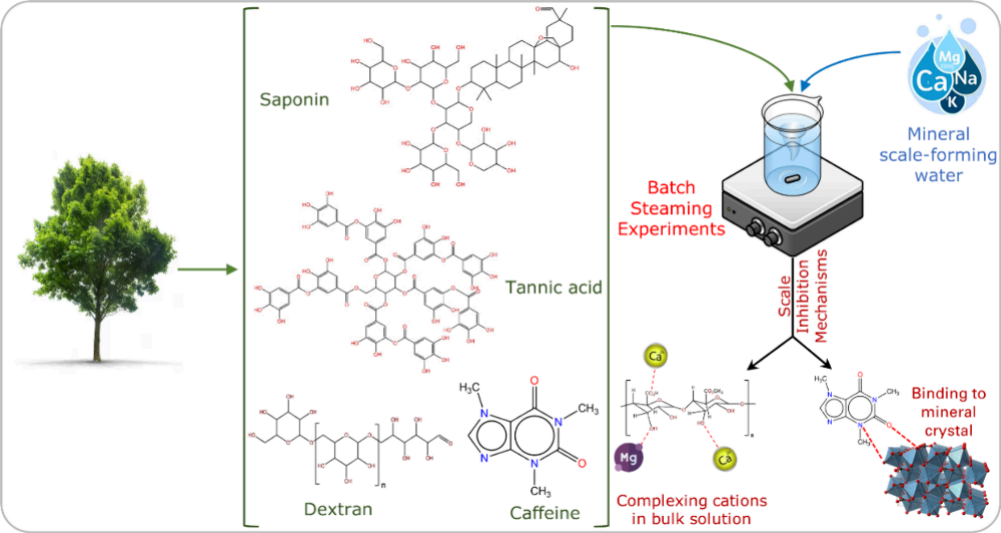

Mineral scale formation reduces the heat transfer efficiency and clogs pipes and valves, increasing power consumption. To address the environmental concerns of conventional scale inhibitors, this paper explores biodegradable and eco-friendly alternatives. It examines the effects of organic additives on calcium (Ca) and magnesium (Mg) scaling in water vaporization. Batch experiments were conducted with potable water and various organic molecules (saponin, caffeine, tannic acid, dextran, citrus pectin, Ficoll 400, and Triton X-100). Saponin showed the highest calcium scale inhibition efficiency (60.9%) followed by caffeine (49.6%) and tannic acid (39.6%), while Ficoll 400, pectin, and Triton X-100 were less effective. For the magnesium scale, caffeine was the most effective (97.4%) followed by saponin (88.6%) and tannic acid (67.1%). Inhibition efficiencies for magnesium-containing scales were generally higher than those for calcium scales. Regarding the inhibition mechanisms, saponin, caffeine, dextran, and tannic acid adsorbed onto mineral crystal growth sites according to the Langmuir model, while pectin, Triton X-100, and Ficoll 400 formed complexes with Ca^2+^ and Mg^2+^ in solution. Needle-like aragonite was the predominant form of calcium carbonate (CaCO_3_) with the most additives, except tannic acid, which produced rhombohedral calcite, and caffeine, which promoted flower-like vaterite CaCO_3_ crystallites. Saponin, caffeine, tannic acid, and dextran are effective, biodegradable, and environmentally friendly inhibitors for mineral scaling.

## 1 Introduction

Mineral scale deposition is a significant challenge encountered in various water processes, including desalination, household appliances, geothermal power plants, and oil and gas production.^1^ These scales, formed primarily from sparingly soluble salts like CaCO_3_, calcium sulfate (CaSO_4_), and magnesium hydroxide (Mg (OH)_2_), can lead to a multitude of problems. One significant consequence of scale formation is the reduction of heat transfer efficiency in cooling systems and the obstruction of pipes. This can significantly impact energy consumption, indirect greenhouse gas emissions, and overall production efficiency.^2,3^ For instance, water evaporation at higher temperatures during the desalination process causes precipitation and deposition of CaCO_3_ and Mg(OH)_2,_ which retard the system’s thermal efficiency. The economic burden of scale deposition is substantial, with estimates suggesting annual costs exceeding £640 million in the United Kingdom, ¥2.4 billion in Japan, and $7.2 billion in the USA.^4^

Traditionally, scale inhibitors have been the primary method for controlling scale formation because they are the most affordable option. These chemicals, typically composed of condensed polyphosphates, organophosphates, and polyelectrolytes, effectively suppress scale development at low dosages (typically below 10 mg/L).^5^ Inhibition occurs through physical mechanisms rather than chemical reactions. Inhibitor molecules attach to growth sites within the scale matrix, causing retardation in nucleation and crystal growth rates. This subsequently results in the formation of distorted crystal structures.^6,7^ Another approach involves complexing with scaling ions in the bulk solution, preventing mineral crystallization.^8^

However, there are growing concerns regarding the environmental impact of these conventional inhibitors. Many are phosphorus-based, such as polyamino polyether methylenephosphonate (PAPEMP) and 1-hydroxyethylidine-1,1-diphosphonic acid (HEDP), having poor biodegradability, potent bioaccumulation, and ecotoxicity.^9,10^ An acute toxicity study conducted on commercially used scale inhibitors containing phosphonates has shown adverse effects on the mortality rate in amphipods.^11^ In addition, phosphonates can break down through hydrolysis to form orthophosphates, which are potential nutrients for algae, and many aquatic systems are controlled by their restricted availability.^10,12^ Hence, the discharge of conventional scale inhibitors in water systems contributes to eutrophication, a process that disrupts aquatic ecosystems, posing severe and long-term effects on maritime living creatures.^13^ Furthermore, conventional antiscalants, such as aminotrimethylene phosphonic acid (ATMP) and polyepoxysuccinic acid (PESA), are not food-safe, which are a significant concern for drinking water desalination systems and household appliances. Therefore, it is crucial for the industry to find natural and environmentally friendly inhibitors to prevent scale formation.^4^

Nowadays, efforts have driven the development of new green inhibitors that, when compared to traditional scale inhibitors, are ecologically friendly and stable. Many green scale inhibitors are derived from organic constituents, such as plant extracts, and have demonstrated voluntary biodegradability with little to no environmental impact. Furthermore, organic polymeric scale inhibitors are relatively cheap as they are derived from affordable and accessible precursors using simple and cost-effective synthetic procedures.^14^ Multiple studies by Abdel-Gaber et al. have investigated the potential use of fig, olive, and *Punica granatum* leaf extracts as biorenewable sourced, alternative calcium mineral scale inhibitors.^15−17^ Abdel-Gaber et al.^17^ proposed that the ellagic acid in the *Punica granatum* hull and leaf extracts forms a complex with Ca^2+^, preventing CaCO_3_ scale deposition and forming a smooth, nonadherent film on steel surfaces. Furthermore, Miksic et al.^18^ have demonstrated that casein-based polymer, soy-based polymer, and polysaccharide from seaweed act as inhibitors for CaCO_3_ mineral scale at relatively high dosages, with efficiencies of 54.2, 16.7, and 16.7%, respectively.

Despite their many advantages, green scale inhibitors require optimal dosing; otherwise, they can be a foulant if concentrations are high. Green scale inhibitors have been shown to increase the biofouling potential in reverse osmosis (RO) systems. Furthermore, the biodegradability component of these inhibitors, although positive in terms of environmental protection, limits their enduring applications, making them an expensive option over time.^14^ Therefore, developing a scale inhibitor that has acceptable biodegradable qualities and high inhibition efficiency at low dosage and is nontoxic, eco-friendly, and affordable will be a prolonged study.

This work explores the potential of natural organic additives as a sustainable solution for inhibiting calcium and magnesium mineral scales. Table 1 shows the organic additives’ chemical structure, molecular weight, and current applications. Saponin, caffeine, tannic acid, dextran, and citrus pectin are natural macro-organic molecules known for their biodegradability, minimal bioaccumulation, and nontoxicity, subject to dosage.^19−22^ Although Triton X-100 is biodegradable by aerobic and anaerobic organisms and UV radiation, it exhibits some toxicity to human and aquatic life with an LD50 (lethal dose for 50% of test subjects) of 1800 mg/kg body weight (Toxicity Category 4).^23,24,25−27^

**Table 1:**
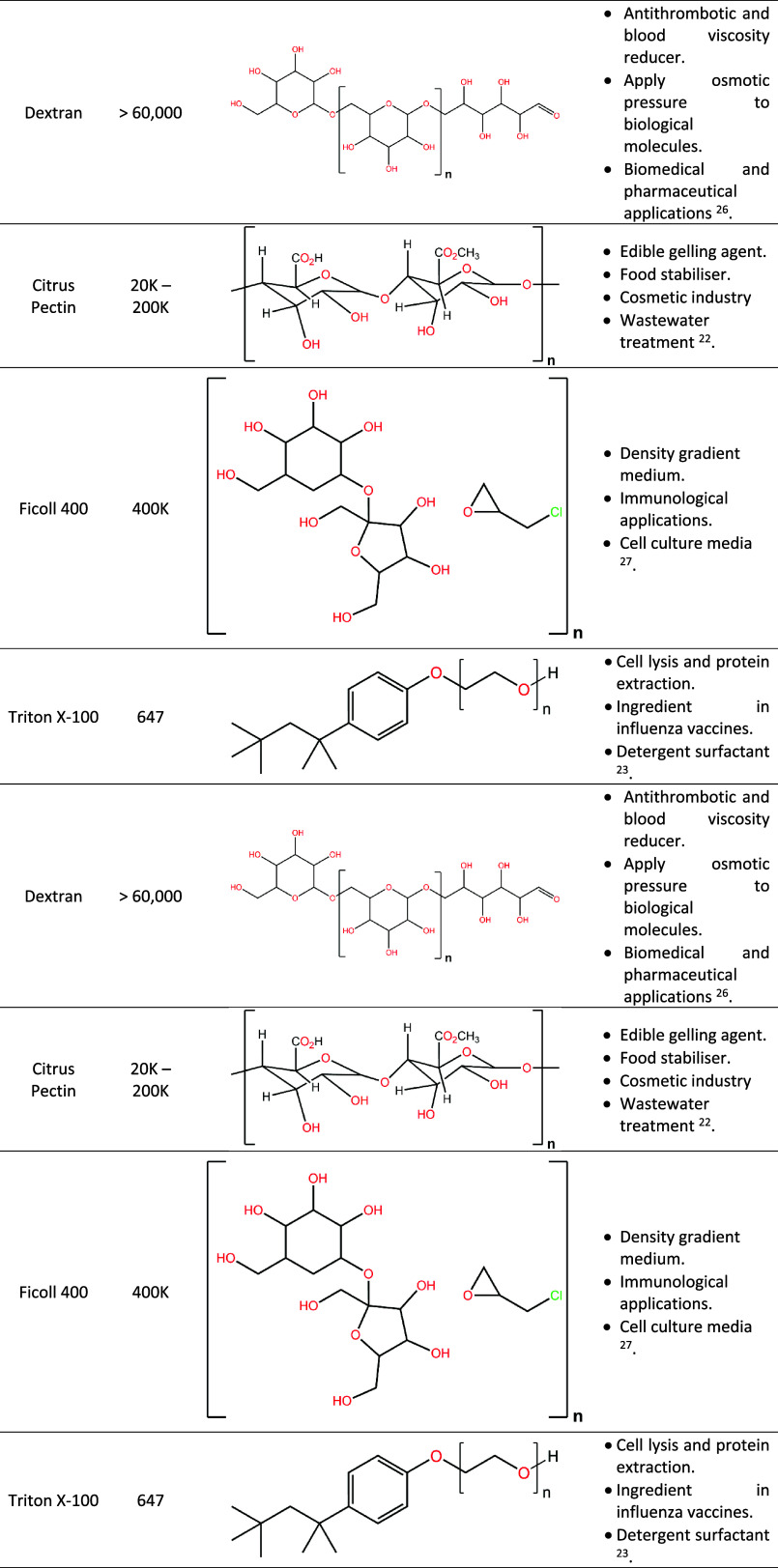
Structure, Molecular Weight, and Applications of the Organic Additives

None of these additives have been studied previously regarding their influence on the precipitation of calcium or magnesium scales. In this work, the inhibition effects of seven organic additives on the mineral precipitation of calcium and magnesium salts from potable water are investigated in a steaming system. The inhibition efficiency is examined in terms of the heating time and temperature function. Furthermore, the adsorption mechanism of the additive molecules on the scale crystal will be evaluated. Finally, the morphological impact of the additives on the precipitated particles will be determined.

## 2 Materials and Methods

### 2.1 Test Solution

Commercially available Evian bottled water was used as a base scale-forming solution due to its high hardness level of 307 mg/L of CaCO_3_ and pH of 7.2, making it the same hardness as the majority of regions in the United Kingdom not situated on chalk beds. The composition of Evian water, as listed on the bottle label, is shown in Table 2. However, upon analysis using inductively coupled plasma optical emission spectroscopy (ICP-OES), variations in the concentrations of certain elements, including Ca^2+^, Mg^2+^, and SO_4_^2–^, were detected in the same batch. For instance, the Ca^2+^ concentration was 90.4 mg/L; Mg^2+^, 30.8 mg/L; and SO_4_^2–^, 10.4 mg/L. Values were averaged from triplicate scans, which were found to be within 10% error on one another. The typical composition on the Evian bottle was used as the basis for error calculation. However, the same batch of Evian bottled water was used in all tests.

**Table 2:** Composition of Evian Drinking Water

**inorganic ion**	mg/L
Ca^2+^	80.0
Mg^2+^	26.0
Na^+^	6.5
K^+^	1.0
Si^4+^	15.0
HCO_3_^–^	360.0
SO_4_^2–^	14.0
Cl^–^	10.0
NO_3_^–^	3.8
dry residue at 180 °C	345.0

Brines for the seven additives were prepared to investigate the effect of organic macromolecules on the precipitation process. For each organic additive, four concentrations, namely, 5, 10, 12.5, and 15 mg/L, were prepared by dissolving the specific additive in Evian water at room temperature. Table 3 displays the details of the organic additives, of which all were supplied by Thermo Scientific Chemicals with no modification or treatment before use. In addition, a quenching solution was used in the sampling to prevent further crystallization in the samples, specifically a solution prepared by mixing 1 g of poly(vinyl sulfonate) (PVS) and 5.71 g of potassium chloride in 1000 mL of deionized water. The pH was then adjusted to a value between 8 and 8.5 using 5 g/L sodium hydroxide solution and a Fisherbrand Hydrus 300 pH meter.

**Table 3:** Organic Additive Information

**additive**	saponin	caffeine	tannic acid	dextran	citrus pectin	Ficoll 400	Triton X-100
State	powder	powder	powder	powder	powder	powder	liquid
Notes	MW: 1080	assay: 99.6% MW: 194.19	MW: 1701	MW: 500k	assay: 74%	assay: ≥98% MW: 400k	MW: 647.2
CAS number	8047-15-2	58-08-2	1401-55-4	9004-54-0	9000-69-5	26873-85-8	9036-19-5
Solubility in water at 25 °C (mg/mL)	50–100	20	250	>30	20–37	100	106

### 2.2 Experimental Setup and Procedure

The mineral precipitation experiments were carried out using a batch crystallizer. This unique setup was developed to mimic a batch steaming process in domestic and industrial systems. The setup consists of a 1000 mL borosilicate glass beaker and hot plate (AREC.X, Thermo Scientific) and thermocouple (Thermo Scientific, UK). In a closed system, solution supersaturation decreases for inversely soluble salts as the temperature increases over time.

The experimental procedure included heating a solution to its boiling temperature under atmospheric pressure. Once the heat source was switched on, solution sampling was conducted every 10 min. The concentration of ionic Ca^2+^ and Mg^2+^ was measured quantitatively. Using a micropipet, 1 mL of the solution sample was mixed with 9 mL of a quenching solution. The solution contents of Ca^2+^ and Mg^2+^ were measured as a function of time using ICP-OES (iCAP 7400 Radial, Thermo Scientific).

The morphology of the precipitated scale was examined using a powder X-ray diffraction (PXRD) using monochromated Cu Kα radiation (λ = 0.1542 nm) on a PANalytical Empyrean series 2 diffractometer. Subsequent analysis of the diffractograms was performed in HighScore Plus with the ICDD’s PDF-2 2012 database. In addition, the samples were examined using a scanning electron microscope (SEM, Zeiss EVO 60) at a pressure of 10^–2^ Pa and an electron acceleration voltage of 20 kV. Before examination, the samples were attached to carbon tape and coated with a 10 nm layer of graphite to enhance contrast and prevent charge accumulation on the sample surface.

## 3 Results and Discussion

### 3.1 Effect of Additive Concentration

**Figure 1:**
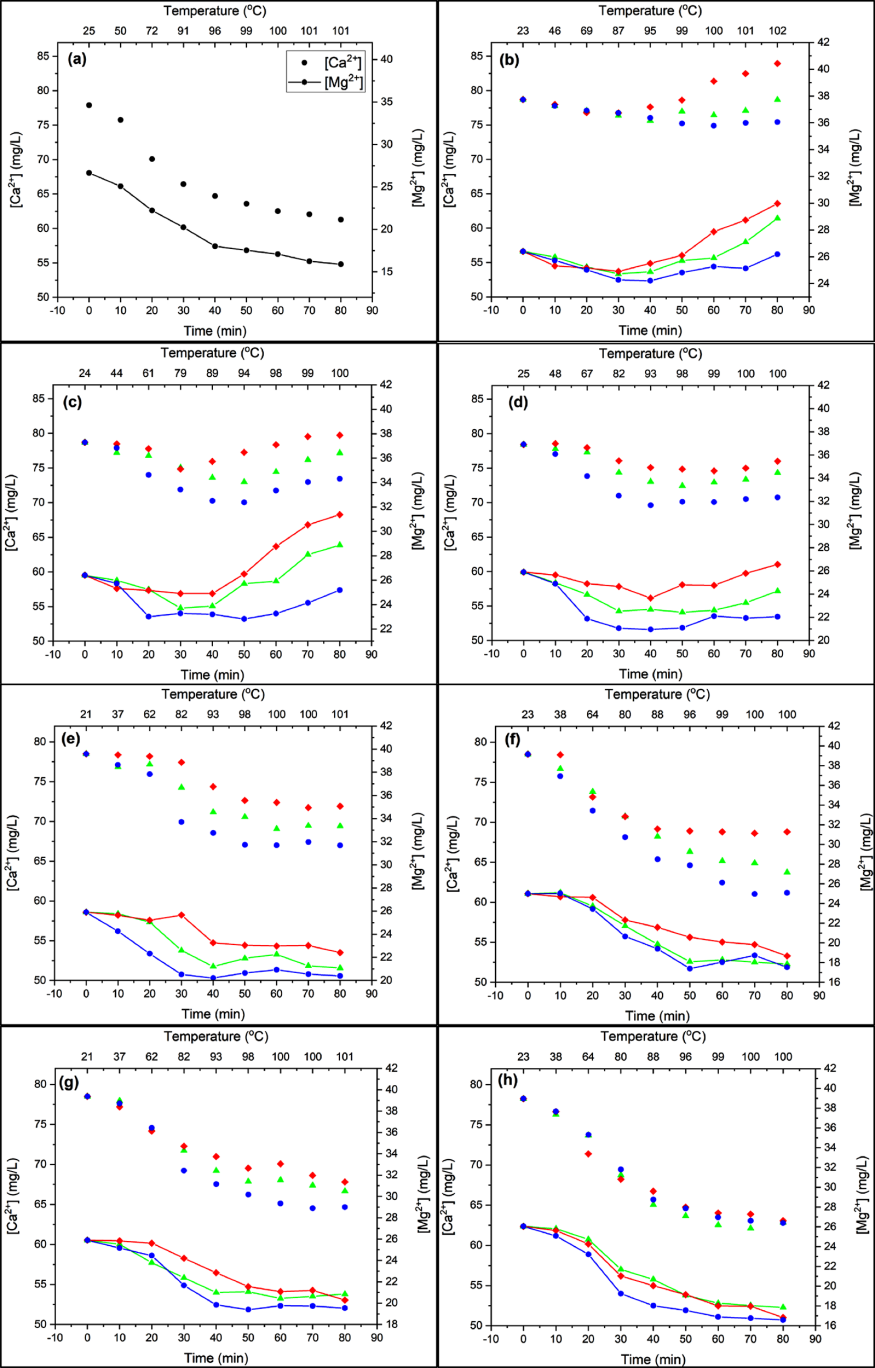
Concentration profile of Ca^2+^ and Mg^2+^ with time and temperature at different concentrations of organic additives: (a) no additive, (b) saponin, (c) caffeine, (d) tannic acid, (e) dextran, (f) citrus pectin, (g) Ficoll 400, and (h) Triton X-100 ([Ca^2+^]: 5 mg/L ●, [Ca^2+^]: 10 mg/L ▲, [Ca^2+^]: 15 mg/L ⧫, [Mg^2+^]: 5 mg/L −●–, [Mg^2+^]: 10 mg/L −▲–, [Mg^2+^]: 15 mg/L −⧫−). Concentrations were measured using ICP-OES.

The effects of seven organic macromolecules were investigated on mineral scaling during the steaming process, as shown in Figure 1. For each additive, four concentrations were tested, namely, 5, 10, 12.5, and 15 mg/L. However, the results of three concentrations for each additive, 5, 10, and 15 mg/L, were presented to avoid complications. The concentrations of Ca^2+^ and Mg^2+^ ions decrease with time and temperature due to the formation of CaCO_3_ and Mg-containing deposits, respectively. Furthermore, Mg^2+^ may be incorporated into the CaCO_3_ crystal, forming crystallites such as dolomite (CaMg(CO_3_)_2_).^28^ As the temperature increases, the evaporation rate of water increases, causing the solution to become supersaturated, with respect to CaCO_3_. The saturation state of the solution decreases thermodynamically with the increased temperature as well as reduced solution volume. As a result of attaining supersaturation, CaCO_3_ will precipitate out into the bulk solution.

Among the tested additives, saponin performs best in inhibiting the consumption rates of Ca^2+^ in the crystallization reactions, as shown in Figure 1b. Saponin retards the consumption of Ca^2+^ and Mg^2+^, especially at the concentration of 15 mg/L. Through the involvement of Ca^2+^ and Mg^2+^ in the crystallization reactions, inorganic precipitate concentrations were found to increase when the temperature reached 87 °C. The increase in concentrations in the bulk solution occurs because the inhibitor restricts the participation of Ca^2+^ and Mg^2+^ in the scale formation reaction. Additionally, continuous heating causes water evaporation, which reduces the solution volume and further increases the concentrations. A similar behavior is observed with other additives, specifically caffeine in Figure 1c and tannic acid in Figure 1d.

Abundant functional groups exist in the saponin structure, including carboxyl, hydroxyl, and carbonyl, in the aglycone and sugar portions. These functional groups, specifically carboxyl and hydroxyl, act by surface adsorption, blocking active growth sites on the crystal surface and preventing further crystal growth.^2,29^ Furthermore, the moderate molecular weight of saponin facilitates the binding of its negatively charged functional groups with the growing crystals.^30^ The lower the molecular weight of an additive is, the higher is its mobility in water, which causes rapid adsorption on the nuclei precursors to inhibit crystal growth.^31^

In the initial 30 min, the temperature increased to 87 °C, leading to the formation of CaCO_3_ and Mg-containing scale entities, which explains the slight drop in the Ca^2+^ and Mg^2+^ concentrations. The minimum concentration is observed at around 40 min, after which the evaporation of water becomes more significant as the temperature approaches 95 °C under atmospheric pressure. This loss of steam leads to an increase in the concentrations. The concentrations subsequently increased due to incorporating saponin into the crystals and increasing the evaporated water volume. The inhibition efficiency was found to increase significantly with additive concentration due to the higher density of negatively charged function groups and increased affinity toward Ca^2+^ and Mg^2+^.^32^

Compared with saponin, caffeine shows less powerful inhibition performance on the mineral scaling, as illustrated in Figure 1c. However, the inhibition mechanism is the same for both additives. Caffeine has the lowest molecular weight among the tested additives, which makes it diffuse faster and interact with the active sites of crystals. It also possesses functional groups with significant affinity to Ca^2+^ and Mg^2+^, such as amine, carbonyl (ketone), and amide. However, the number of functional groups in the caffeine structure is much lower than that in saponin. The amount of functional groups relative to the Ca^2+^ and Mg^2+^ concentrations is crucial for inhibiting the bulk precipitation.^33^

Figure 1d displays the Ca^2+^ and Mg^2+^ concentration profiles as functions of time and temperature in the presence of tannic acid. Although the hydrophilic functional groups are abundant in tannic acid, they show moderate inhibition effects compared to caffeine and saponin. The reduced impact is attributed to the relatively large molecular weight and decreased affinity of the hydroxyl group due to temperature increase.^2^ The higher temperatures mean increased thermal energy to the system, leading to greater molecular kinetic energy and therefore disruption of the intermolecular forces responsible for binding. Dextran is another additive that showed moderate inhibition effects, as shown in Figure 1e. According to the concentration profiles of Ca^2+^ and Mg^2+^, dextran interacts differently with fouling species at high concentrations. At 15 mg/L dextran, the induction time of bulk precipitation is prolonged to ∼30 min at 80 °C. This implies that the dextran additive at higher concentration complexes with the Ca^2+^ and Mg^2+^ ions in the bulk solution, leaving less available ions for growth/build up on the active sites of the crystal surface. Afterward, the inhibition efficiency decreases with temperature, which enhances the kinetic energy of the cations and improves the crystal growth rate.^34^

Furthermore, the impact of dextran on the cation concentrations is insignificant at low concentrations. As a result, dextran is a suitable inhibitor for low- to moderate-temperature processes. The main functional group in dextran is hydroxyl, which offers an easy point for chemical conjugation with cations such as Ca^2+^.^35^ However, the absence of carboxyl groups and relatively high molecular weight (>60,000 g/mol) make dextran not an ideal candidate for surface adsorption and crystal growth inhibition. Hydroxyl groups have previously been found to have a lower surface-binding capacity than carboxyl groups due to their inability to form hydrogen bonds.^36^

Citrus pectin showed an insignificant impact on either the precipitation rate or the induction time, as shown in Figure 1f. This implies that citrus pectin has a low capability of complexing with the cations in the bulk solution or incorporating crystals to inhibit the growth. The relativity poor inhibition effects of citrus pectin are attributed to its high molecular weight (20,000–200,000 g/mol) and relatively low density of negatively charged functional groups compared to the additives tested above. However, the precipitation rate of CaCO_3_ was found to reduce by 12.2% with increased citrus pectin loading in the solution from 5 to 15 mg/L. Due to the structure of the inhibitor, the larger quantity of carboxyl and hydroxyl functional groups (highlighted in red in Table 1) are available to interact with Ca^2+^ with increased inhibitor concentration.

Ficoll 400 showed almost the same performance as citrus pectin in the inhibition of Ca^2+^ and Mg^2+^ mineral formation, as illustrated in Figure 1g. Ficoll 400 is a highly branched polymer with a molecular weight of ∼400 K g/mol, which results in poor diffusion characteristics that aid in interacting with crystal surfaces. Moreover, the Ficoll 400 structure contains only hydroxyl and epoxide groups, which are less powerful than carboxyl, carboxylate, amine, or amide when complexed with cations.^1^ Regarding the additive loading, insignificant inhibition impacts were observed when the loading changed from 5 to 15 mg/L, especially on the Mg^2+^ concentration. However, at 15 mg/L Ficoll 400, prolongation in the induction of Mg crystallization was observed.

The last additive in Figure 1h is Triton-X100, which shows a slight improvement in inhibiting crystallization compared to the case without any additive in Figure 1a. The poor inhibition properties of Triton-X100 are attributed to the low density of negatively charged groups. It also has a linear structure, which is less efficient in binding to the mineral crystals or complexing with free cations in the bulk. Reddy and Hoch^37^ suggested that a cyclic and rigid antiscalant structure is much more efficient toward scaling compared to linear molecules. The role of van der Waals interactions with hydrophobic alkyl groups in the surface-binding process might be insignificant.

**Figure 2:**
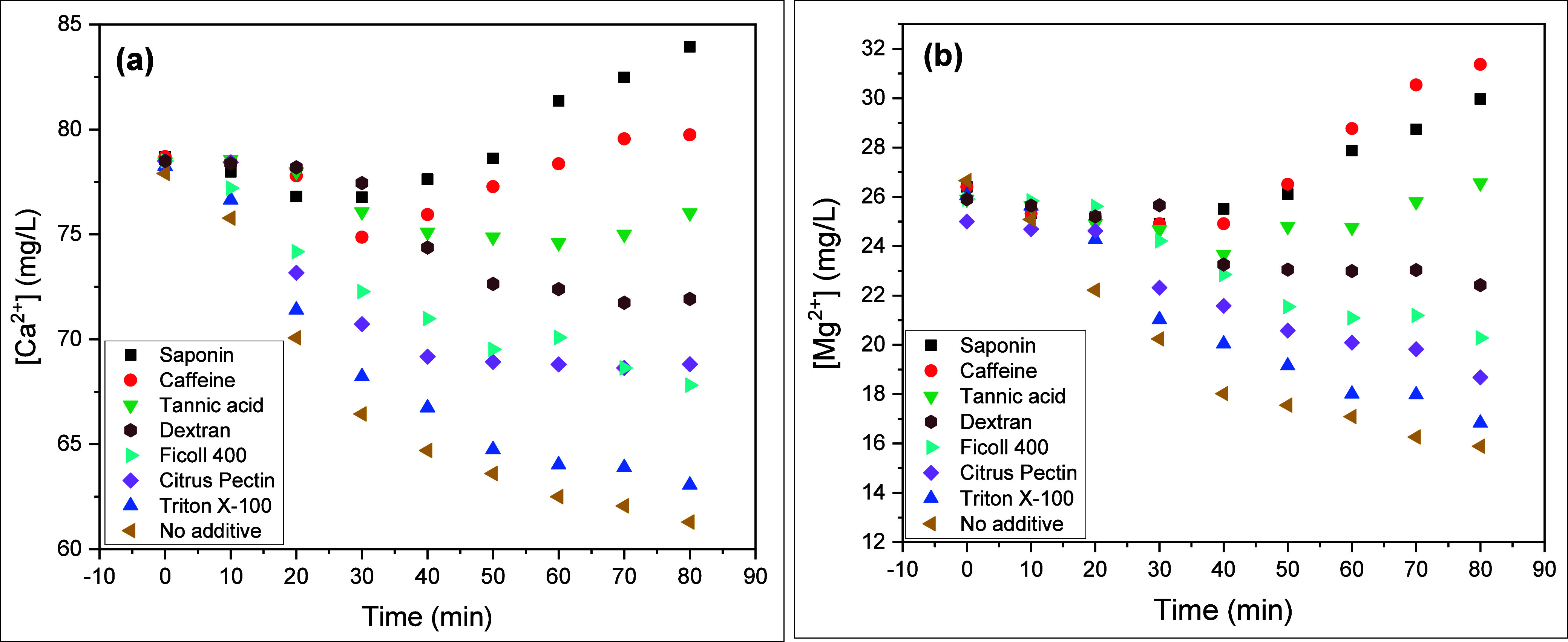
Effect of organic additives on the precipitation of calcium (a) and magnesium (b) minerals during the steaming process. Concentrations were measured using ICP-OES.

Figure 2 shows the effects of the organic additives on the concentration profile of Ca^2+^ and Mg^2+^ at an additive concentration of 15 mg/L. For Ca^2+^, saponin has the highest inhibition effect followed by caffeine and tannic acid. Caffeine showed slight superiority over saponin in inhibiting Mg mineral scaling, especially after 40 min of the experiment. Triton-X100 exhibits the poorest performance for both cations where the concentrations of Mg^2+^ and Ca^2+^ are too close to the additive-free case at constant time and temperature.

**Figure 3:**
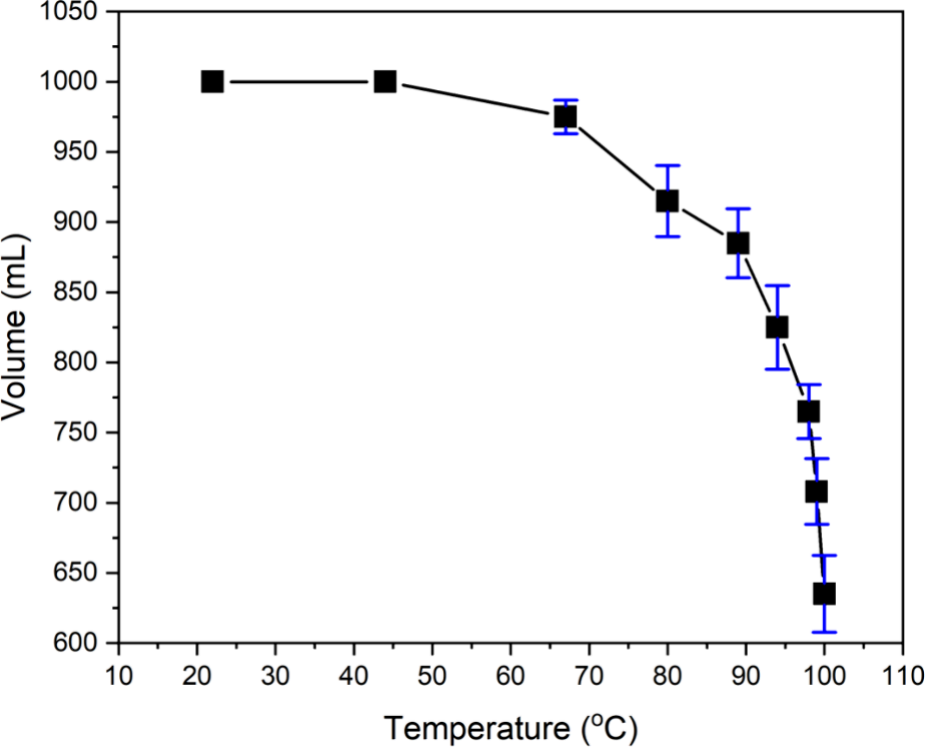
Variation of the evaporated volume of water with temperature.

The inhibition efficiency of each additive was calculated to compare the impact and temperature resistance of each additive. However, the solution volume is not constant during the steaming process. The evaporation rate and evaporated volume were measured with the temperature in several experiments. Figure 3 shows the change in the evaporated volume with the temperature. The following expression (eq 1) determines the inhibition efficiency.
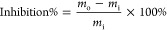
1where *m*_o_ and *m*_i_ are the mass of a foulant (Ca^2+^ or Mg^2+^) in the absence and presence of organic additives, respectively.

**Figure 4:**
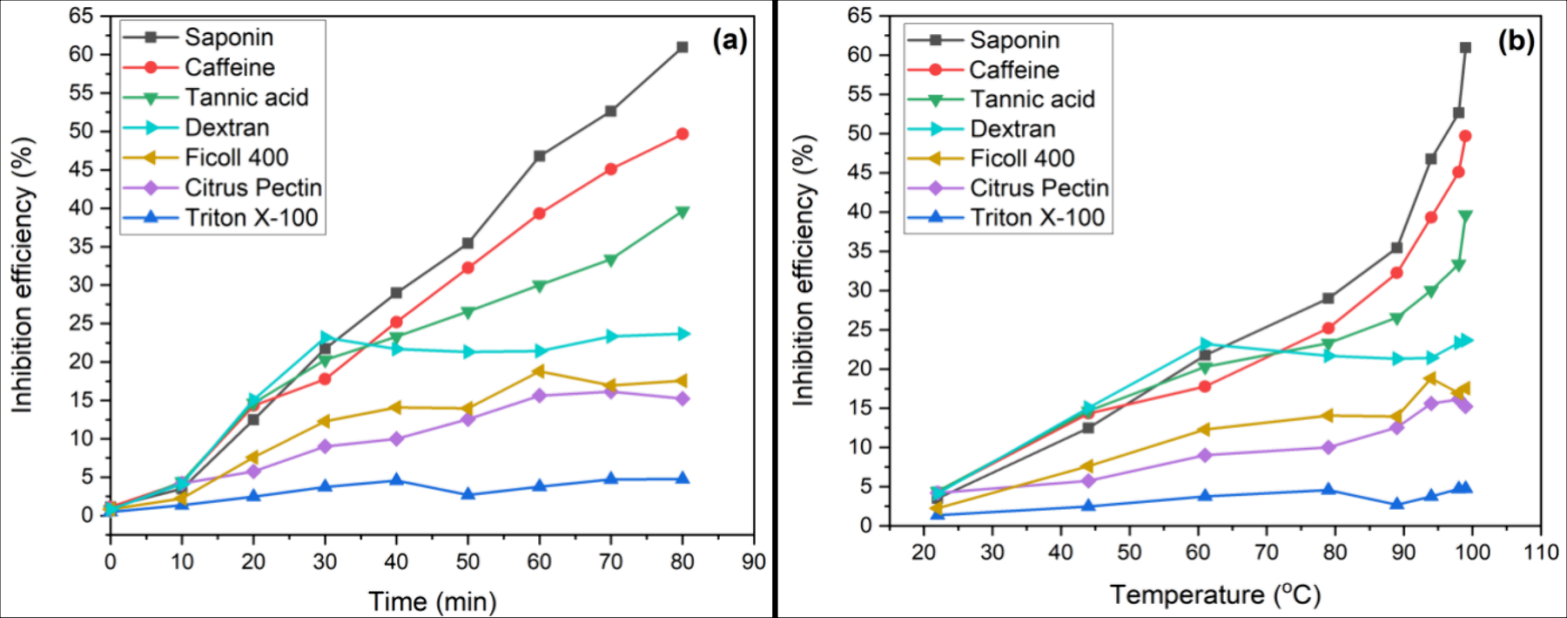
Efficiency of the 15 mg/L organic additives in inhibiting Ca^2+^ scale formation with respect to time (a) and temperature (b).

**Figure 5:**
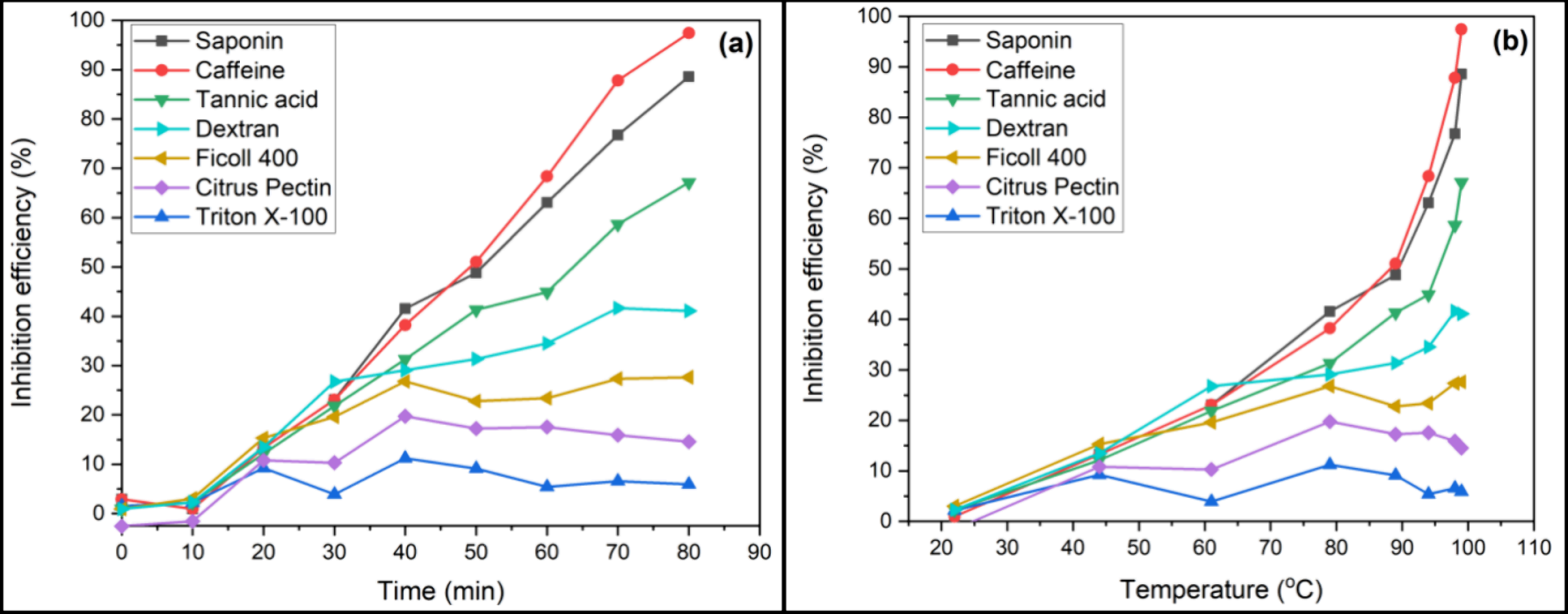
Efficiency of the 15 mg/L organic additives in inhibiting Mg^2+^ scale formation with respect to time (a) and temperature (b).

The efficiencies of tannic acid, saponin, and caffeine in the inhibition of Ca^2+^ and Mg^2+^ scale formation increase with time and temperature, as displayed in Figures 4 and 5. Saponin and caffeine show the most significant efficiencies in inhibiting Ca^2+^ and Mg^2+^ scale formation at 60.9 and 97.4%, respectively. The inhibition efficiency for the Mg^2+^ scale is generally higher than that for the Ca^2+^ scale. Li et al.^38^ reported that the inhibition efficiency of the PAP-1 antiscalant [a poly(aspartic acid) and poly(carboxylic acid)] for the Mg-containing scale is slightly higher than that for CaCO_3_. This difference can be attributed to the fact that Ca^2+^ is larger in Mg^2+^ due to increasing electron shielding, where the radii are recorded as 99 and 79 pm, respectively. As a result, tannic acid, saponin, and caffeine molecules are able to interact with smaller ions compared to large ones efficiently.

However, dextran, Ficoll 400, citrus pectin, and Triton X-100 experience a decrease in the inhibition efficiency with a temperature increase above 60 °C for both Ca- and Mg-containing scales (Figures 4b and 5b). The reduction in the efficiency with temperature increase is because of the enhanced crystallization reaction kinetics,^39^ reduced adsorption rate of additive molecules on scale crystal, and increased kinetic energy of the ions.^40^

### 3.2 Adsorption Mechanism

**Figure 6:**
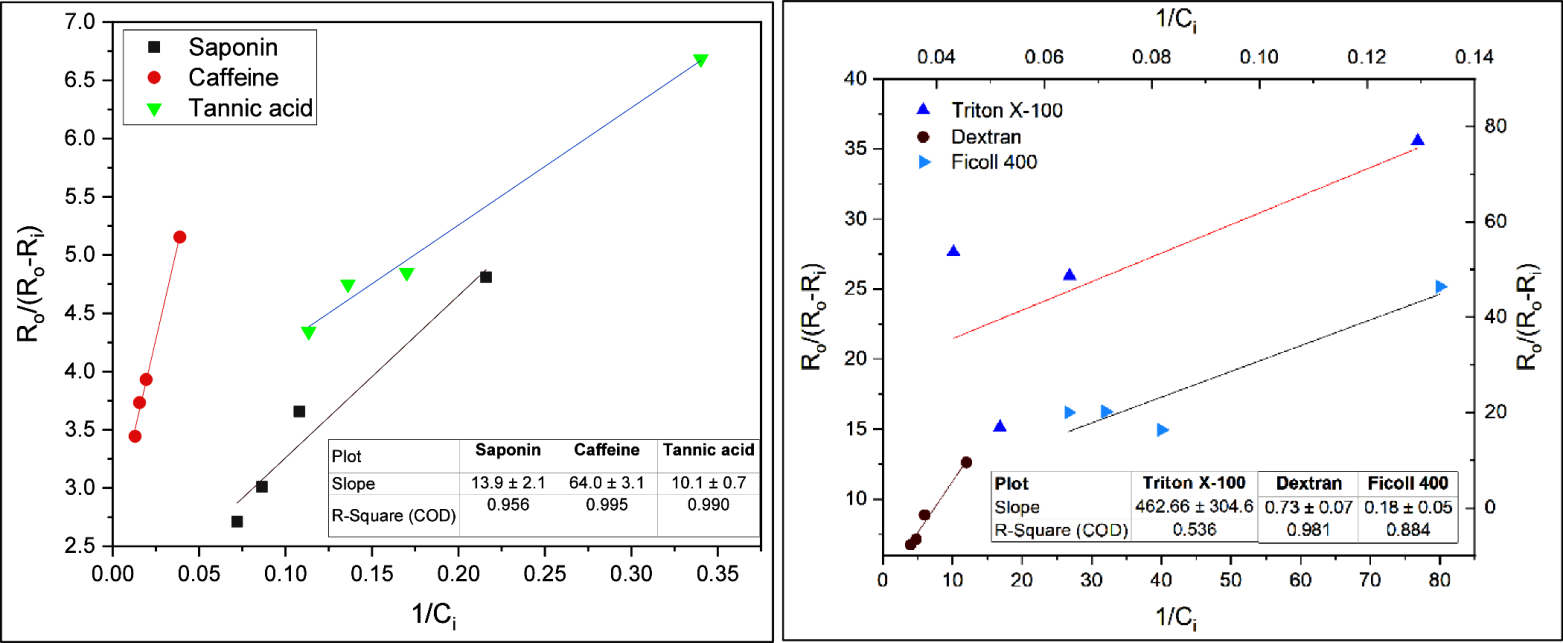
Kinetics of CaCO_3_ crystal growth in the presence of different organic additives based on a Langmuir adsorption model.

Adsorption of inhibitors at active sites on crystal surfaces can lead to a reduction in the rate of surface-controlled growth. The inhibition observed in this study of tannic acid, saponin, and caffeine was most likely due to blocking active growth sites on crystals rather than binding to cations in the solution due to several factors, such as carboxyl groups and molecular weight. Most scaling inhibitors reduce reaction rates and could be interpreted using the Langmuir adsorption isotherm,^41,42^ which assumes that the reduction in rate results from the adsorption of inhibitor molecules at growth sites. The Langmuir model has been widely used to explain the reduction in growth rates of many sparingly soluble salts in the presence of inhibitors.^1,43,44^ The model assumes reversible adsorption at a finite number of identical sites on the surface, forming a monolayer, which dictates that the adsorption and desorption rates at equilibrium are equal. If biopolymers adsorb reversibly at crystal surfaces, the measured growth rates as a function of inhibitor concentration (*C*_i_) can be examined for Langmuir coverage, as expressed below:
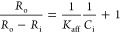
2where *R*_o_ and *R*_i_ are the precipitation rates in the absence and presence of inhibitor, respectively, and *K*_aff_ is the affinity constant that is a measure of the affinity of the adsorbate (inhibitor) for the adsorbent (crystal surface). *K*_aff_ is the ratio of the specific rate constant of adsorption to that of desorption (*K*_aff_ = *k*_ads_/*k*_des_). The data were plotted according to eq 2 to check the validity of the models, as shown in Figures 6 and7. The excellent linearity of saponin, caffeine, dextran, and tannic acid (*R*^2^ > 0.95) suggests that the inhibitory effects of these additives are due to adsorption at active growth sites. The orientation of the macromolecule could be affected by the carboxyl groups in the additive backbone, causing a significant increase in the degree of association with calcium ions in the crystal lattice of CaCO_3_.^45^ Carboxyl and hydroxyl groups are highly electronegative, enabling them to form stable bonds with Ca^2+^ on CaCO_3_ crystal surfaces.^46^ The oxygen atom within the carboxyl group carries a stronger negative charge, creating a strong electrostatic attraction between the carboxyl group and Ca^2+^.^47^

**Figure 7:**
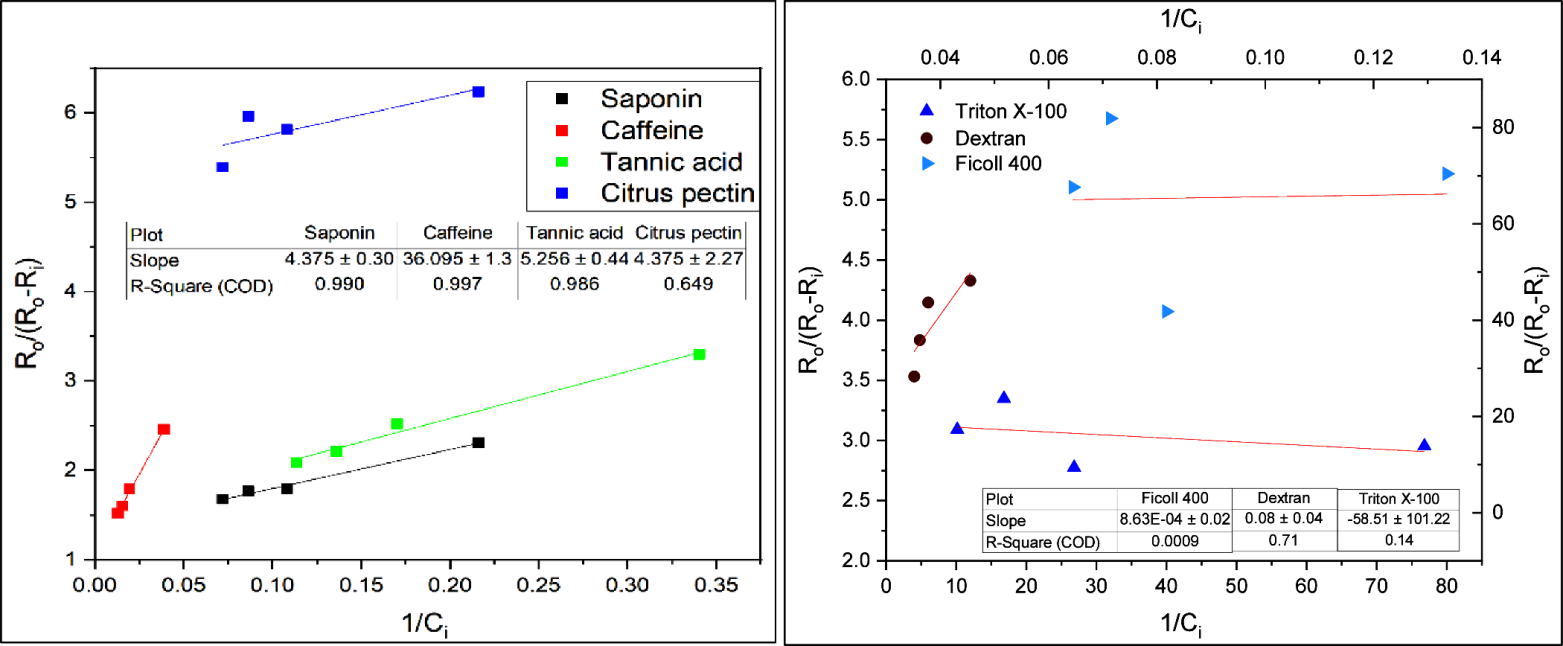
Kinetics of Mg-containing scale crystal growth in the presence of different organic additives based on a Langmuir adsorption model.

However, the plots for Triton X-100 and Ficoll 400 show poor linearity, with *R*^2^ values of 0.536 and 0.884, respectively, indicating that their inhibition effect is likely due to interactions with Ca^2+^ in the bulk solution rather than adsorption onto the scale crystals. This suggests that due to their high molecular weight and lack of key functional groups, these additives do not follow the Langmuir adsorption model. Instead, they likely inhibit scale formation by complexing with Ca^2+^ and Mg^2+^ ions in the bulk solution. The citrus pectin data of CaCO_3_ inhibition are not plotted due to negative *y*-axis values resulting from a higher growth rate than the base case (inhibitor-free).

The plots in Figure 7 show that saponin, caffeine, and tannic acid follow the Langmuir model in inhibiting Mg-based precipitates by blocking the growth of active sites. Unlike its performance with CaCO_3_, the Langmuir model is invalid for dextran, with an Mg-containing scale showing a low *R*^2^ value of 0.71. Triton X-100, citrus pectin, and Ficoll 400 exhibit poor affinity toward Mg-containing scale crystals. For the additive plots with high *R*^2^, the calculated affinity constants toward CaCO_3_ and Mg-containing precipitates were determined from the slope, as listed in Table 4. Among the tested additives, dextran showed the highest affinity toward the CaCO_3_ scale at 1.37 × 10^6^ due to the high density of hydroxyl functional groups.^48^ Generally, the affinity of additives toward the Mg-containing precipitates is higher than that for CaCO_3_. This affinity variation might be due to differences in crystal structure and surface energy. For instance, MgCO_3_ (magnesite) has a hexagonal crystal lattice, aragonite CaCO_3_ has an orthorhombic crystal lattice, and calcite CaCO_3_ has a rhombohedral crystal.

**Table 4:** Affinity Constants for Various Inhibitors on the Inhibition of CaCO_3_ and Mg-Containing Scale in dm^3^/mol

**Additive**	**CaCO**_**3**_	**Mg-containing scale**
Saponin	7.34 × 10^4^	2.29 × 10^5^
Caffeine	1.56 × 10^4^	2.77 × 10^4^
Tannic acid	9.93 × 10^4^	1.90 × 10^5^
Dextran	1.37 × 10^6^	

### 3.3 Effect of the Organic Additives on the Crystal Morphology

**Figure 8:**
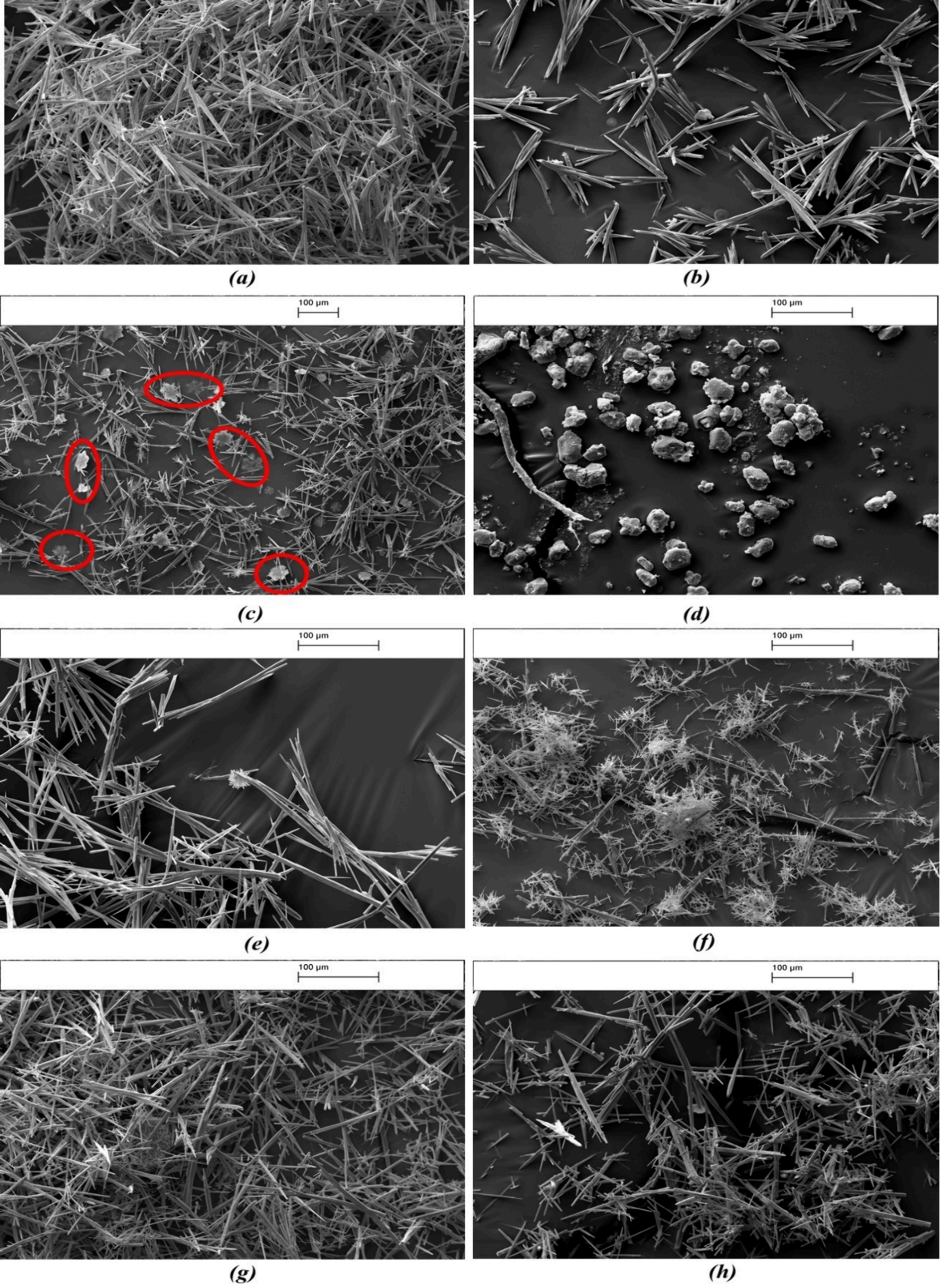
SEM analysis of the CaCO_3_ crystal structure for (a) no additive and in the presence of (b) saponin, (c) caffeine, (d) tannic acid, (e) dextran, (f) citrus pectin, (g) Ficoll 400, and (h) Triton X-100 with a concentration of 15 mg/L.

**Figure 9:**
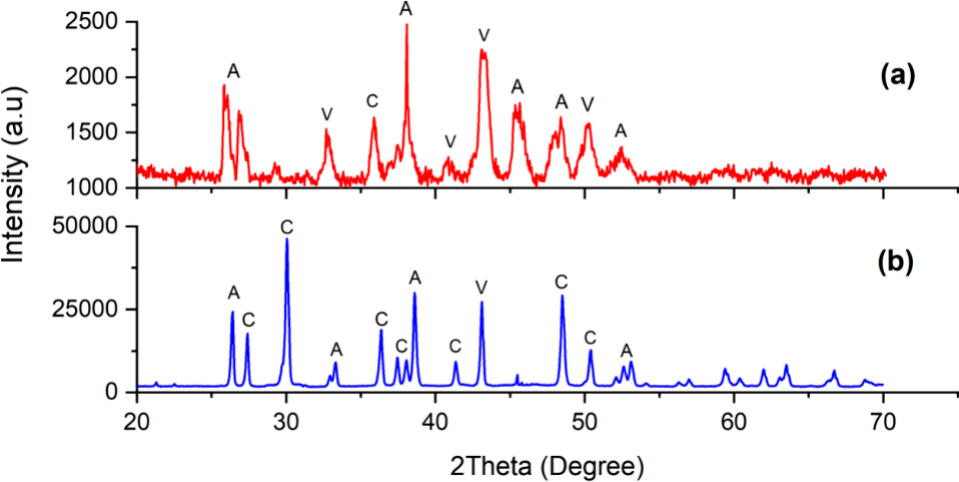
XRD patterns for the precipitated minerals with 15 mg/L of (a) caffeine and (b) tannic acid (A: aragonite, C: calcite, and V: vaterite).

Scanning electron microscopy (SEM) micrographs in Figure 8 show the effects of organic additives on the crystal morphology. The needle-like aragonite is the dominant polymorphic phase of calcium carbonate formed in the presence of most additives except tannic acid. The majority of crystals precipitated are rhombohedral calcite under the effect of tannic acid, as shown in Figure 8d. The hydroxyl group in the additive enhances the phase transition process from aragonite to calcite.^49^ Tannic acid contains a relatively high density of phenolic hydroxyl groups compared with other additives. The powder X-ray diffraction (XRD) analysis in Figure 9 reveals that calcite is the predominant polymorphic phase of CaCO_3_ in the presence of tannic acid. Aragonite is also observed in the tannic acid samples but will be less available. Regarding caffeine effects, the amine group has been suggested to promote the formation of vaterite CaCO_3_ and slow the transformation of vaterite into a more stable calcite over time, a change from a hexagonal to rhombohedral crystal structure.^50,51^ The transformation of vaterite to calcite involves the dissolution of vaterite, releasing ions into the solution, which then reprecipitate on the surface of calcite crystals, leading to the layer-by-layer growth of calcite.^52^ Besides the needle-like aragonite, flower-like vaterite crystallites are also observed, as shown in Figure 8c (highlighted in red). The XRD analysis also confirms that caffeine induces the formation of vaterite crystals in bulk precipitation.

The dimensions of a minimum of 20 crystals in each sample were measured by SEM images. The mean crystal size in each sample is shown in Table 5. As seen, tannic acid inhibition results in the smallest scale particles, with an average size of 49.6 ± 4.5 μm, followed by saponin and caffeine. Citrus pectin and Ficoll 400 showed an insignificant reduction in crystal size compared with the base case (no additive). Both additives demonstrate limited effectiveness in inhibiting scale crystal growth, leading to a minimal reduction in crystal size. However, this method is semiquantitative, relying on a limited number of samples, which provides only rough estimates of crystal sizes. The variability in the findings is considerable, with the exception of those for dextran and citrus pectin.

**Table 5:** Mean Crystal Size from Different Additive Experiments

**Additive**	Saponin	Caffeine	Tannic acid	Dextran	Citrus pectin	Ficoll 400	Triton X-100	No additives
Size (μm)	86.0 ± 9.5	94.3 ± 9.0	49.6 ± 4.5	109.2 ± 5.5	133.2 ± 5.9	126.8 ± 8.1	110.0 ± 10.0	132.3 ± 13.5

## 4 Conclusions

The inhibitory effects of organic additives on the mineral scaling of calcium and magnesium salts were investigated during a steaming process. The bulk precipitation from potable water was conducted by using a batch system in different concentrations of saponin, caffeine, tannic acid, dextran, citrus pectin, Ficoll 400, and Triton X-100. The concentrations of Ca^2+^ and Mg^2+^ were measured using ICP-OES as a function of heating time and system temperature. The concentration data were used to evaluate the adsorption mechanism of the organic molecules on the scale crystals. Furthermore, the morphology of precipitated minerals was examined by using SEM and XRD.

The results showed that concentrations of Ca^2+^ and Mg^2+^ decrease with temperature and time due to a crystallization reaction followed by an increase at the higher temperatures due to solution evaporation and volume reduction. For the Ca scale inhibition, saponin exhibits the highest inhibition efficiency with 60.9% followed by caffeine (49.6%) and tannic acid (39.6%), while Ficoll 400, citrus pectin, and Triton X-100 show the poorest efficiency. Regarding Mg-based salt formation, caffeine has the best performance with 97.4% followed by saponin (88.6%) and tannic acid (67.1%). Overall, the inhibition efficiencies on the Mg-containing scale formation were much higher than those of the Ca scale.

In terms of the inhibition mechanisms, saponin, caffeine, dextran, and tannic acid are absorbed in the active growth sites of the mineral crystals, following the Langmuir adsorption model. Nevertheless, citrus pectin, Triton X-100, and Ficoll 400 seem to form a complex with Ca^2+^ and Mg^2+^ in the bulk solution. Regarding the morphological investigations, needle-like aragonite is the dominant phase of CaCO_3_ formed with most additives, except tannic acid, which causes rhombohedral calcite to precipitate and form the smallest scale particles among the tested additives, with an average size of 49.6 ± 4.5 μm. Furthermore, caffeine was the only additive that promoted the formation of flower-like vaterite CaCO_3_ due to its amine functionality. Overall, saponin, caffeine, tannic acid, and dextran are approved as biodegradable, environmentally friendly, and natural organic inhibitors for mineral scaling in potable water systems and, as such, should be seen as an option to replace current inhibitors such as phosphorus-based inhibitors.
